# Supplementary material for: Spider Silk Fibroin Protein Heterologously Produced in Rice Seeds Reduce Diabetes and Hypercholesterolemia in Mice
Source: Plants (Basel). 2020 Sep 28;9(10):1282. doi: 10.3390/plants9101282 (PMC7650732; doi:10.3390/plants9101282)
Supplement: Supplementary file 1 [file plants-09-01282-s001.zip › Supplementary Table 1.docx]

**Table 1.** Composition of experimental diets (g/100 g diet)

| **Components** | **C** | **FC** | **DC-23** | **DC-46** | **TRL3-23** | **TRL3-46** | **TRL5-23** | **TRL5-46** |
| --- | --- | --- | --- | --- | --- | --- | --- | --- |
| Casein | 20 | 20 | 20 | 20 | 20 | 20 | 20 | 20 |
| Corn starch | 49.949 | 45.949 | 22.975 | 0 | 22.975 | 0 | 22.975 | 0 |
| Sucrose | 10 | 10 | 10 | 10 | 10 | 10 | 10 | 10 |
| Corn oil | 10 | 7 | 7 | 7 | 7 | 7 | 7 | 7 |
| Lard | 0 | 7 | 7 | 7 | 7 | 7 | 7 | 7 |
| Cellulose | 5 | 5 | 5 | 5 | 5 | 5 | 5 | 5 |
| Vitamin mix | 1 | 1 | 1 | 1 | 1 | 1 | 1 | 1 |
| Mineral mix | 3.5 | 3.5 | 3.5 | 3.5 | 3.5 | 3.5 | 3.5 | 3.5 |
| Choline bitart | 0.25 | 0.25 | 0.25 | 0.25 | 0.25 | 0.25 | 0.25 | 0.25 |
| DL-methionine | 0.3 | 0.3 | 0.3 | 0.3 | 0.3 | 0.3 | 0.3 | 0.3 |
| BTH | 0.001 | 0.001 | 0.001 | 0.001 | 0.001 | 0.001 | 0.001 | 0.001 |
| Dong-Jin rice | 0 | 0 | 22.975 | 45.949 | 0 | 0 | 0 | 0 |
| Transgenic rice L3 | 0 | 0 | 0 | 0 | 22.975 | 45.949 | 0 | 0 |
| Transgenic rice L5 | 0 | 0 | 0 | 0 | 0 | 0 | 22.975 | 45.949 |
| Total | 100 | 100 | 100 | 100 | 100 | 100 | 100 | 100 |
