# Supplementary material for: Spider Silk Fibroin Protein Heterologously Produced in Rice Seeds Reduce Diabetes and Hypercholesterolemia in Mice
Source: Plants (Basel). 2020 Sep 28;9(10):1282. doi: 10.3390/plants9101282 (PMC7650732; doi:10.3390/plants9101282)
Supplement: Supplementary file 1 [file plants-09-01282-s001.zip › Supplementary Table 2.docx]

Table 2 Body weight and liver weight

| **Group** | **Body weight gain (g/four weeks)** | **Liver weight (g/mouse)** |
| --- | --- | --- |
| C | 35.03±2.93 | 1.37±0.02 |
| FC | 38.06±6.00 | 1.58±0.09 |
| DC-23 | 36.80±4.61 | 1.50±0.05 |
| TRL3-23 | 35.40±3.80 | 1.40±0.03** |
| TRL5-23 | 36.00±4.60 | 1.43±0.06 |
| DC-46 | 35.66±3.14 | 1.49±0.07 |
| TRL3-46 | 35.86±3.63 | 1.37±0.05* |
| TRL5-46 | 34.04±3.04 | 1.38±0.02* |
