# Supplementary figures and images for: Spider Silk Fibroin Protein Heterologously Produced in Rice Seeds Reduce Diabetes and Hypercholesterolemia in Mice
Source: Plants (Basel). 2020 Sep 28;9(10):1282. doi: 10.3390/plants9101282 (PMC7650732; doi:10.3390/plants9101282)

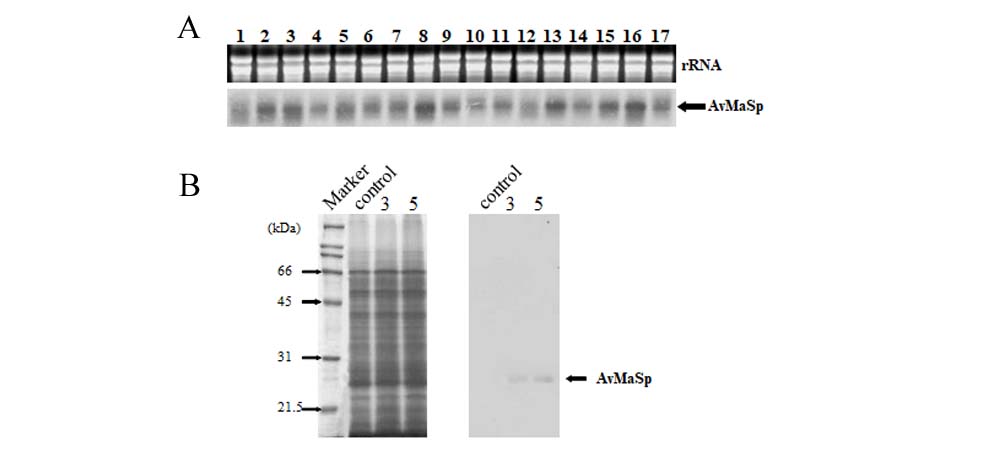

Supplement: Supplementary file 1 [file plants-09-01282-s001.zip › Supplementary Fig 1.jpg]

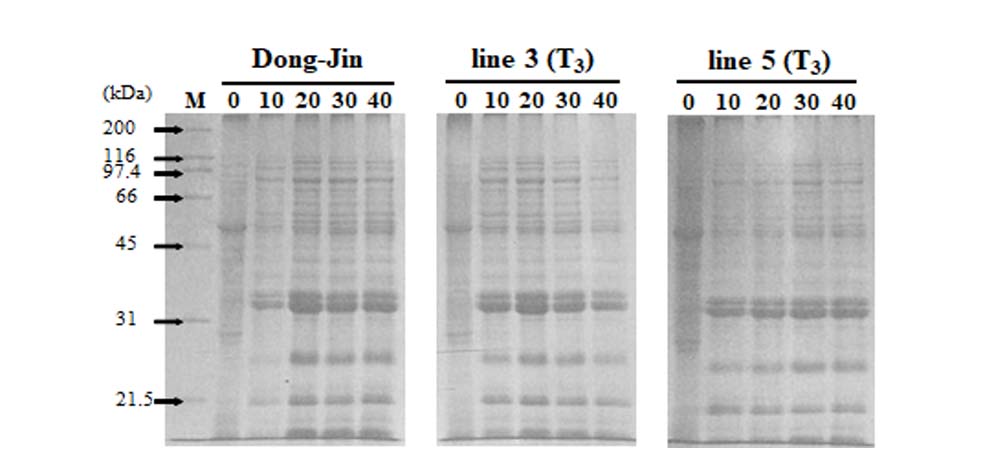

Supplement: Supplementary file 1 [file plants-09-01282-s001.zip › Supplementary Fig 2.jpg]
